# Effects of Red Sorghum-Derived Deoxyanthocyanidins and Their O-β-D-Glucosides on E-Cadherin Promoter Activity in PC-3 Prostate Cancer Cells

**DOI:** 10.3390/molecules29081891

**Published:** 2024-04-21

**Authors:** Nathalie Mora, Maxence Rosa, Mohamed Touaibia, Luc J. Martin

**Affiliations:** 1 UMR408 INRA–UAPV, SQPO, Qualim, University Avignon, Campus Jean-Henri Fabre, Pôle Agrosciences, 301, Rue Baruch de Spinoza, 84911 Avignon, France; nathalie.mora@univ-avignon.fr (N.M.); maxence.rosa@univ-avignon.fr (M.R.); 2 Chemistry and Biochemistry Department, Université de Moncton, Moncton, NB E1A 3E9, Canada; mohamed.touaibia@umoncton.ca; 3 Biology Department, Université de Moncton, Moncton, NB E1A 3E9, Canada

**Keywords:** 3-deoxyanthocyanidin, 3-deoxyanthocyanin, PC-3 cells, transcription factors, E-cadherin

## Abstract

Although much less common than anthocyanins, 3-Deoxyanthocyanidins (3-DAs) and their glucosides can be found in cereals such as red sorghum. It is speculated that their bioavailability is higher than that of anthocyanins. Thus far, little is known regarding the therapeutic effects of 3-DAs and their O-β-D-glucosides on cancer, including prostate cancer. Thus, we evaluated their potential to decrease cell viability, to modulate the activity of transcription factors such as NFκB, CREB, and SOX, and to regulate the expression of the gene *CDH1*, encoding E-Cadherin. We found that 4′,7-dihydroxyflavylium chloride (P7) and the natural apigeninidin can reduce cell viability, whereas 4′,7-dihydroxyflavylium chloride (P7) and 4′-hydroxy-7-O-β-D-glucopyranosyloxyflavylium chloride (P3) increase the activities of NFkB, CREB, and SOX transcription factors, leading to the upregulation of *CDH1* promoter activity in PC-3 prostate cancer cells. Thus, these compounds may contribute to the inhibition of the epithelial-to-mesenchymal transition in cancer cells and prevent the metastatic activity of more aggressive forms of androgen-resistant prostate cancer.

## 1. Introduction

The colors of flowers, fruits, vegetables, and cereals can be attributed to their content in anthocyanins and vary from red to blue according to pH, self-association, and interactions with phenolic copigments, such as flavonols, flavones, and hydroxycinnamic acids, and metal ions (Al^3+^, Fe^3+^, Mg^2+^) [1,2,3]. Anthocyanins were found to be a subclass of flavonoids present in many flowers, fruits, and vegetables, as well as in fruit-based beverages, notably red wine. Because of their natural coloring properties, anthocyanins make a major contribution to the quality and consumer appeal of food. They can also be associated with the health benefits of a diet rich in plant products [4].

**Scheme 1:**
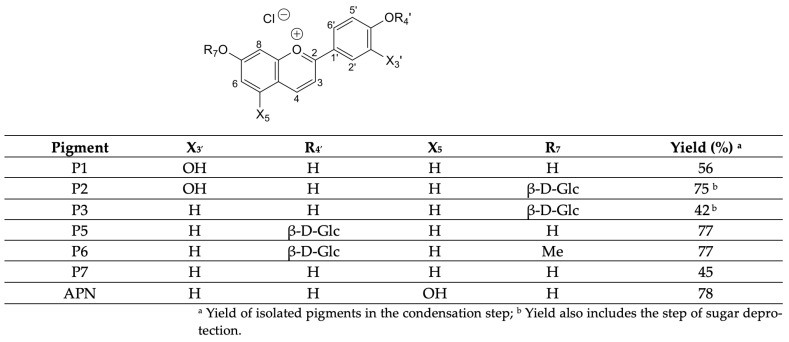
Pigments investigated.

Cereals such as red sorghum are an important source of 3-deoxyanthocyanidins (3-DA) and their glucosides [5]. 3-Deoxyanthocyanidins are more stable than anthocyanidins because of their absence of a C3-OH group, which plays an essential role in their degradation [6]. Under slightly acidic to neutral conditions, 3-DAs have more intense and more stable colors than common anthocyanins. Indeed, the absence of the OH group on C3 makes these pigments considerably less sensitive to the addition of water at C2 [7,8] (see Scheme 1), which makes them more resistant to irreversible chemical degradation and reversibly leads to colorless hemiketal and chalcone forms [9].

These pigments (3-DA) are also promising for their potential health benefits, expressed through cell-specific antioxidant effects [10,11,12]. Moreover, their bioavailability could be expected to be better than that of anthocyanins. Indeed, 3-deoxyanthocyanidins are probably less subject to catabolism in the gastrointestinal tract. To date, little is known about the therapeutic effects of 3-DAs and their O-β-D-glucosides on cancer, including prostate cancer.

In our recent work, we reported the chemical synthesis of 3-DAs and their O-β-D-glucosides (Scheme 1), including the typical sorghum red natural pigments apigeninidin (APN, 4′,5,7-trihydroxyflavylium) and luteolinidin (LTN, 3′4′,5,7-tetrahydroxyflavylium) [13,14]. However, the possible therapeutic effects of these 3-DAs and their O-β-D-glucosides on cancer, including prostate cancer, are not well known.

Mainly diagnosed in men over 50, prostate cancer has favorable 5-year survival rates as a result of early detection and the availability of curative surgery. However, progression to a more aggressive form resistant to androgen deprivation and with increased metastatic activity is responsible for the majority of deaths associated with prostate cancer. Thus, alternative treatments for prostate cancer are worth considering and could involve the use of 3-DAs and their O-β-D-glucosides.

Transcription factors are DNA-binding proteins with a sequence-specific DNA-binding domain and an activation/repression domain, which interacts with multiple coregulators, contributing to the recruitment of RNA polymerase II. Transcription factors can act as activators or repressors of transcription, depending on the cofactors and chromatin modulators recruited [15]. Changes in the activity of transcription factors are associated with the development or prevention of various diseases, including prostate cancer. In addition to the androgen receptor, other transcription factors are involved in regulating various cancer properties associated with prostate tumorigenesis. Among these, the cyclic AMP response element (CRE)-binding protein (CREB), the SRY-box transcription factors (SOX), and the nuclear factor kappa-light-chain-enhancer of activated B cells (NFkB) have been characterized as regulators of gene expression influencing prostate cancer progressions [16,17,18].

Among the different types of molecules that keep cells together, cadherins play an important role in the formation of adherens junctions, thus mediating calcium-dependent cell-cell adhesion [19]. During the development of aggressive cancer, the epithelial-to-mesenchymal transition (EMT), characterized by a change in the expression from E-cadherin (*CDH1*) to N-cadherin (*CDH2*), enables cancer cells to acquire invasive and metastatic properties [20,21]. Indeed, the potential for prostate cancer invasiveness and metastasis is suppressed by the over-expression of *CDH1* [22]. Interestingly, the activation of CREB transcription factors by protein kinase A has been associated with a mesenchymal-to-epithelial transition as a result of increased *CDH1* expression [23]. NFkB activation has been linked to the inhibition of *CDH1* expression [24], thus promoting EMT of prostate cancer cells. Members of the SOX family can be divided into transcriptional activators and repressors [25]. Hence, depending on which SOX factors are expressed in cancer cells, they may have an activating or inhibitory effect on *CDH1* expression.

The aim of this work is to study the structure–activity relationships of synthesized pigments, including 3-DAs and their O-β-D-glucosides, on cell viability and gene regulation related to the epithelial-to-mesenchymal transition in the androgen-resistant prostate cancer cell line PC-3.

## 2. Results

### 2.1. Effects of Synthesized Pigments on Cell Viability

The chemical structures of the synthesized pigments assessed for their effects on the viability of PC-3 prostate cancer cells and the expression of *CDH1*-related genes are presented in Scheme 1.

**Figure 1:**
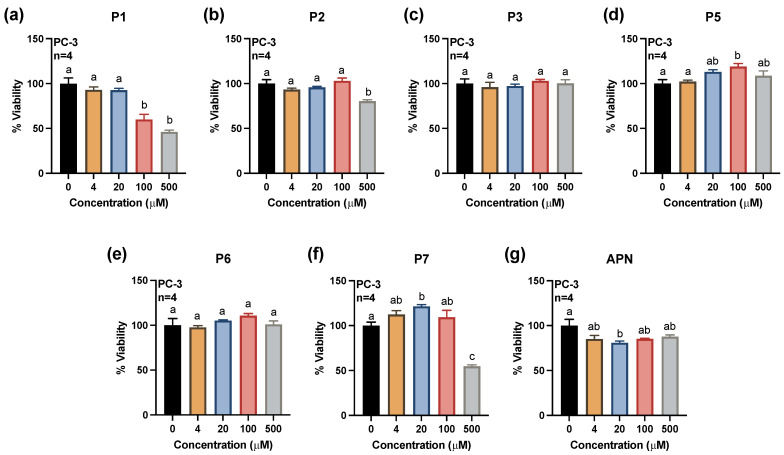
Viability of PC-3 prostate cancer cells in response to treatments with the indicated molecules. Cells were incubated in the absence or presence of increasing concentrations (0, 4, 20, 100, 500 µM) of the indicated molecules for 24 h, followed by the determination of cell viability as described in the Material and Methods Section. Results for the viability of PC-3 cells following treatments with P1 (**a**), P2 (**b**), P3 (**c**), P5 (**d**), P6 (**e**), P7 (**f**), and APN (**g**) are presented. The experiments were repeated four times, and the results are presented as percent cell viability over control (0 μM, DMSO only) (±SEM). Statistical comparisons were performed using a one-way ANOVA followed by a Holm-Sidak multiple comparison test, where different letters denote significant differences (*p* < 0.05).

To better assess the structure–activity relationship of the synthesized pigments and their anti-cancer properties against prostate cancer, the viability of androgen-resistant PC-3 prostate cancer cells was first evaluated following 24 h treatments with increasing concentrations of the molecules (Figure 1). Interestingly, treatment with the P1 molecule resulted in a reduction in PC-3 cell viability by 40% at 100 μM and by 54% at 500 μM (Figure 1a). The P2 molecule resulted in a modest reduction in cell viability by 20% at 500 μM, while the P7 molecule decreased cell viability by 46% at 500 μM (Figure 1b,f). The OH at position 3′, as in the P1 molecule, seems to be decisive for the reduction in cell viability. Glycosylation at position 7 (P2 molecule) and the absence of the hydroxyl group at position 3′ (P7 molecule) do not have a significant effect on the reduction in the viability of PC-3 cells (Figure 1b,f).

Compared with the P5 molecule, the addition of a methyl moiety at position 7 has no influence on cell viability, as demonstrated with the P6 molecule for all tested concentrations (Figure 1e).

Although the APN molecule appears to decrease the viability of PC-3 cells at all the concentrations evaluated, only the concentration of 20 μM resulted in a significant reduction of 19% (Figure 1g). As shown by the effect of APN, the presence of a hydroxyl at position 5 is more decisive for a better reduction in PC-3 cell viability.

### 2.2. Influences of Synthesized Pigments on the Expression of E-Cadherin-Related Genes

**Figure 2:**
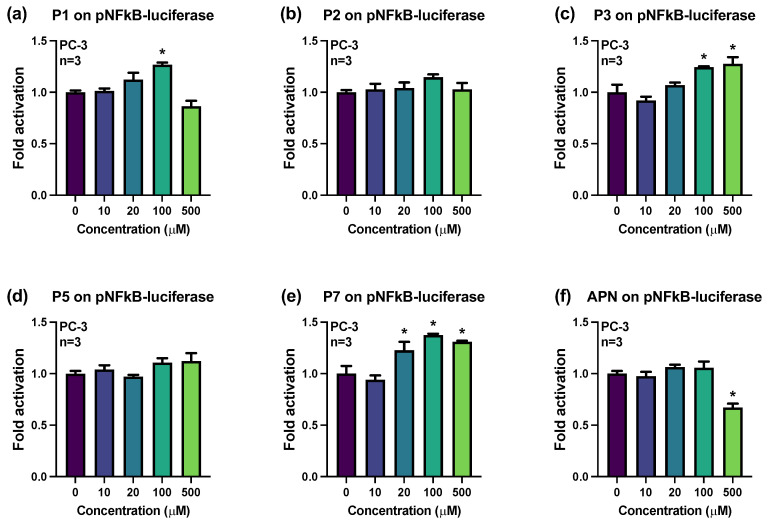
Effects of the indicated molecules on the activity of NFkB transcription factors. PC-3 prostate cancer cells were transfected with a luciferase plasmid reporter harboring four consensus NFkB regulatory elements upstream of the gene encoding *Firefly* luciferase. Cells were treated with increasing concentrations of the indicated molecules for 6 h. Results for the activation of the NFκB transcription factors following treatments of PC-3 cells with P1 (**a**), P2 (**b**), P3 (**c**), P5 (**d**), P7 (**e**), and APN (**f**) are presented. Results are presented as fold activation over the control (DMSO only) (±SEM). A one-way ANOVA was used to analyze data according to the concentrations of the indicated molecules, followed by Dunnett’s multiple comparison test for significant differences compared to the control (* *p* < 0.05).

To better define the potential influence of synthesized pigments on the regulation of gene expression in prostate cancer cells, different reporter plasmid constructs harboring the consensus regulatory elements for NFkB, CREB, or SOX were transfected in PC-3 cells followed by treatments for 24 h with increasing concentrations of the molecules. Interestingly, treatment with the P1 molecule increased the activity of NFkB transcription factors by 1.27 folds at 100 μM (Figure 2a), while the P3 molecule resulted in increases by 1.24 folds at 100 μM and by 1.28 folds at 500 μM (Figure 2c). In addition, the P7 molecule increased the activity of NFkB transcription factors by more than 1.23 folds at concentrations from 20 to 500 μM (Figure 2e). The presence or absence of the hydroxyl or the D-glycosyl moieties at positions 3′ or 7 leads to increased activity of NFkB transcription factors. Oppositely, the APN molecule bearing only a hydroxyl moiety at position 5′ was the only pigment that significantly decreased the activity of NFkB transcription factors by 33% at 500 μM (Figure 2f).

**Figure 3:**
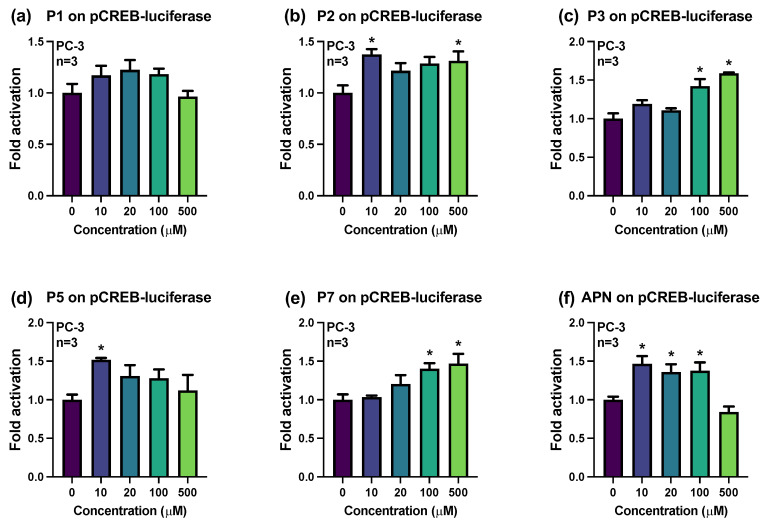
Effects of the indicated molecules on the activity of CREB transcription factors. PC-3 prostate cancer cells were transfected with a luciferase plasmid reporter harboring four consensus CREB regulatory elements upstream of the gene encoding *Firefly* luciferase. Cells were treated with increasing concentrations of the indicated molecules for 6 h. Results for the activation of the CREB transcription factors following treatments of PC-3 cells with P1 (**a**), P2 (**b**), P3 (**c**), P5 (**d**), P7 (**e**), and APN (**f**) are presented. Results are presented as fold activation over the control (DMSO only) (±SEM). A one-way ANOVA was used to analyze data according to the concentrations of the indicated molecules, followed by Dunnett’s multiple comparison test for significant differences compared to the control (* *p* < 0.05).

Regarding the influence of synthesized pigments on the activity of CREB transcription factors, PC-3 prostate cancer cells were transfected with a reporter plasmid construct harboring CREB consensus regulatory elements followed by treatments for 24 h with increasing concentrations of the molecules (Figure 3). Interestingly, treatment with the P2 molecule increased the activity of CREB transcription factors by 1.37 and 1.31 folds at 10 and 500 μM, respectively (Figure 3b). The activity of CREB transcription factors was also increased by more than 1.42 folds following treatments with the P3 molecule at concentrations of 100 and 500 μM (Figure 3c), as well as by 1.52 folds in response to treatment with the P5 molecule at 10 μM (Figure 3d). Treatment with the P7 molecule also increased the activity of CREB transcription factors by more than 1.40 folds at concentrations of 100 and 500 μM (Figure 3e), whereas treatment with the APN molecule resulted in an increase by more than 1.36 folds at concentrations ranging from 10 to 100 μM (Figure 3f).

In contrast to the effect on the activity of NFkB transcription factors, molecules P2, P3, P5, P7, and APN increased the activity of CREB transcription factors.

**Figure 4:**
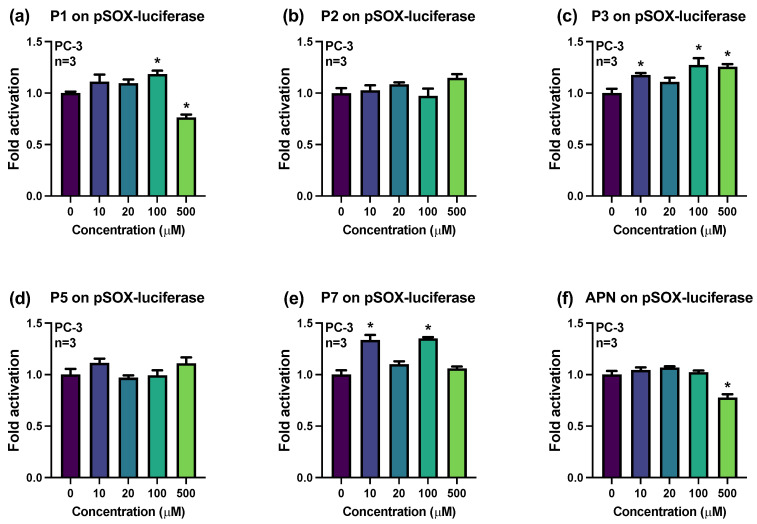
Effects of the indicated molecules on the activity of SOX transcription factors. PC-3 prostate cancer cells were transfected with a luciferase plasmid reporter harboring four consensus SOX regulatory elements upstream of the gene encoding *Firefly* luciferase. Cells were treated with increasing concentrations of the indicated molecules for 6 h. Results for the activation of SOX transcription factors following treatments of PC-3 cells with P1 (**a**), P2 (**b**), P3 (**c**), P5 (**d**), P7 (**e**), and APN (**f**) are presented. Results are presented as fold activation over the control (DMSO only) (±SEM). A one-way ANOVA was used to analyze data according to the concentrations of the indicated molecules, followed by Dunnett’s multiple comparison test for significant differences compared to the control (* *p* < 0.05).

To evaluate the influence of the synthesized pigments on the activity of SOX transcription factors, PC-3 prostate cancer cells were transfected with a reporter plasmid construct harboring SOX consensus regulatory elements followed by treatments with increasing concentrations of the molecules for 24 h (Figure 4). This SOX DNA regulatory element can interact with SOX4, 5, 8, 9, 10, 12, 13, 14, 15, 17, or 18. According to RNA-Seq data from PC-3 cells [26], the SOX members SOX4, 9, 12, 13, 15, 17, and 18 are expressed in this cell model and can be redundant in regulating pSOX-luc activity. Although treatment with the P1 molecule increased the activity of SOX transcription factors by 1.18 folds at 100 μM, such activity was decreased by 24% at 500 μM (Figure 4a). The activity of SOX transcription factors was increased by more than 1.18 folds following treatments with the P3 molecule at concentrations of 10, 100, and 500 μM (Figure 4c), as well as by more than 1.34 folds in response to treatment with the P7 molecule at 10 or 100 μM (Figure 4e). However, treatment with the APN molecule decreased the activity of SOX transcription factors in PC-3 cells by 22% at 500 μM (Figure 4f).

As with the effect on the activity of NFkB transcription factor, molecules P2, P3, P5, and P7 increased the activity of SOX transcription factors. As for NFkB activity, APN is the only molecule that decreases the activity of SOX transcription factors.

**Figure 5:**
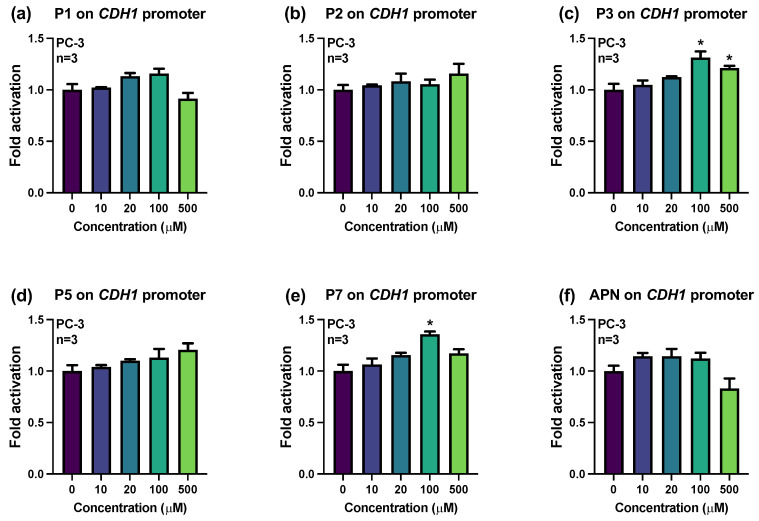
Effects of the indicated molecules on the activation of the *CDH1* promoter. PC-3 prostate cancer cells were transfected with a luciferase plasmid reporter harboring the human −670 to +92 bp *CDH1* promoter upstream of the gene encoding *Firefly* luciferase. Cells were treated with increasing concentrations of the indicated molecules for 6 h. Results for the activation of the *CDH1* promoter following treatments of PC-3 cells with P1 (**a**), P2 (**b**), P3 (**c**), P5 (**d**), P7 (**e**), and APN (**f**) are presented. Results are presented as fold activation over the control (DMSO only) (±SEM). A one-way ANOVA was used to analyze data according to the concentrations of indicated molecules, followed by Dunnett’s multiple comparison test for significant differences compared to the control (* *p* < 0.05).

Since the activity of the *CDH1* gene promoter can be influenced by the presence and changes in the activity of the NFkB, CREB, and SOX transcription factors [27], we evaluated the potential effects of synthesized pigments on the regulation of the human −670 to +92 bp *CDH1* promoter region (Figure 5). Interestingly, treatment with the P3 molecule activated the *CDH1* promoter by 1.31 and 1.21 folds at 100 and 500 μM, respectively (Figure 5c), while treatment with the P7 molecule resulted in an increase of 1.36 folds at 100 μM (Figure 5e). The P7 molecule or its glycosylated analog (P3) at position 7 were the only molecules with an activating effect on this gene. The presence of a hydroxyl at the 3′ and 5′ positions and a glycosyl at the 4′ position had no effect on this gene’s activation.

### 2.3. Physicochemical Properties and Drug-likeness Prediction

As shown in Table 1, the ADME (adsorption, distribution, metabolism, and excretion) properties of APN and the investigated analogs (P1–P7) were predicted using SwissADME [28]. APN, analog P1, and analogs P3–P6 are all in agreement with Lipinski’s Rule of five [29]. All analogs will have high bioavailability since they have a predicted topological surface area (TSA) below 140 (Table 1). Analogs P1, P6, and P7 and APN will have high gastrointestinal absorption (GIA) and no ability to cross the blood–brain barrier except for molecule P7 (Table 1). Analog P2 is the only one to violate Lipinski’s Rule of five [29] as its number of hydrogen bond acceptors (HBA) is greater than 5 and its TPS is greater than 140 (Table 1).

## 3. Discussion

Regarding the viability of PC-3 prostate cancer cells in response to treatments with the synthesized pigments, the P1 molecule, bearing a hydroxyl at position 3′, appears to be the most promising with reduced viability at concentrations of 100 to 500 μM. However, P1 only increases the activity of the NFkB transcription factor while having no effect on *CDH1* promoter activity. Among the synthesized pigments activating the transcription factors NFkB, CREB, and SOX in PC-3 cells, P7 and its glycosylated analog P3 are promising as these molecules also increase *CDH1* promoter activity. Increased *CDH1* expression in cancer cells reduces their ability to undergo epithelial–mesenchymal transition [30] and consequently, their metastatic potential. Interestingly, the expression of *CDH1* may be activated by SOX17, as suggested in esophageal cancer cells [31]. In addition, SOX17 is also highly expressed in prostate cancer [32].

The constitutive activation of NFkB transcription factors is commonly observed in different types of cancer. During prostate cancer progression, NFkB activation promotes cell survival, tumor invasion, metastatic activity, and chemoresistance [33]. Moreover, constitutive NFkB activation is associated with loss of androgen receptor expression and with an androgen-resistant phenotype of prostate cancer [34]. Thus, the inhibition of NFkB activity by APN could promote the treatment of highly aggressive, chemotherapy-resistant prostate cancers.

Interestingly, APN also decreases the activity of SOX transcription factors in PC-3 prostate cancer cells. However, the inhibitory effects of APN on the activities of NFkB and SOX factors do not translate into a modulation of *CDH1* promoter activity. This suggests that APN may inhibit prostate cancer development by influencing the activity of signaling pathways or transcript factors involved in the tumorigenesis, proliferation, invasion, and/or metastatic activity of cancer cells. Interestingly, APN reduced the viability of HL-60 and HepG2 human cancer cells by more than 40% following treatments of 48 h with concentrations from 100 to 200 μM [35]. In addition, extracts containing APN from *Sorghum bicolor* decreased the viability of different cancer cells, including DU145 and LNCap prostate cancer cells [36]. Furthermore, one extract induced apoptosis of A549 lung cancer cells by decreasing the phosphorylation of the signal transducer and activator of transcription 3 (STAT3) transcription factor and the activation of caspase 3 [36]. However, STAT3 is not expressed in PC-3 cells (*our personal observations*). Thus, further investigation is needed to elucidate the mechanism of action responsible for APN cytotoxicity in prostate cancer cells.

The regulation of *CDH1* expression by the transcription factors SOX, CREB, and NFkB is supported by the presence of DNA regulatory elements enabling their recruitment to the −670 to +92 bp *CDH1* promoter region. Indeed, the *CDH1* promoter contains two SOX DNA regulatory elements at −307 and −177 bp and two NFkB DNA regulatory elements at −73 and −30 bp. In addition, more than 12 potential DNA regulatory elements can be found for CREB1 recruitment in the −670 to +92 bp *CDH1* promoter region. However, the direct protein–DNA interactions between these transcription factors and the promoter region of *CDH1* remain to be confirmed. Moreover, the distinction between the anti-cancer properties associated with the activation of the SOX, CREB, and NFkB transcription factors and their pro-cancer properties remains to be more clearly defined through further research. It is also possible that the actions of the synthesized pigments on the activation of *CDH1* expression are independent of the activation of the transcription factors SOX, CREB, and NFkB, and rather involve the activation or inhibition of another transcription factor yet to be characterized. However, a correlation between the activation of CREB1 and increased expression of *CDH1* in normal prostate luminal cells has been proposed by others [37]. Also, SOX1 increases the expression of *CDH1*, leading to the suppression of cell growth and invasiveness of cervical cancer [38]. Furthermore, the addition of several weak activations or repressions of transcription factors can have a significant biological impact on the regulation of target genes, including *CDH1*, in prostate cancer cells.

The correlation between *CDH1* expression and prostate cancer cell viability is not straightforward and requires further investigation to be fully elucidated [39]. In this study, only the synthesized pigment P7 is reported to increase *CDH1* promoter activity and decrease PC-3 cell viability. However, these results are obtained at different concentrations. Thus, these findings suggest that the molecular mechanisms responsible for the increase in *CDH1* promoter activity by synthesized pigments are different from those involved in the reduction in prostate cancer cell viability.

Since the expression of *CDH1* is known to limit the invasion and metastasis of human cancer cells [40], it would be interesting to assess the influence of synthesized pigments such as 3-DAs and their O-β-D-glucosides on the migration of PC-3 prostate cancer cells. This experiment will be carried out as part of our future research. Since the activation of NFkB is linked to the inhibition of *CDH1* expression [24], it would be interesting to evaluate the effects of synthesized pigments on the expression of other NFkB-regulated genes in addition to *CDH1* in PC-3 cells. These results would enable us to better define the anticancer properties of red sorghum-derived deoxyanthocyanidins and their O-β-D-glucosides.

## 4. Materials and Methods

### 4.1. Synthesis of Compounds

**Scheme 2:**
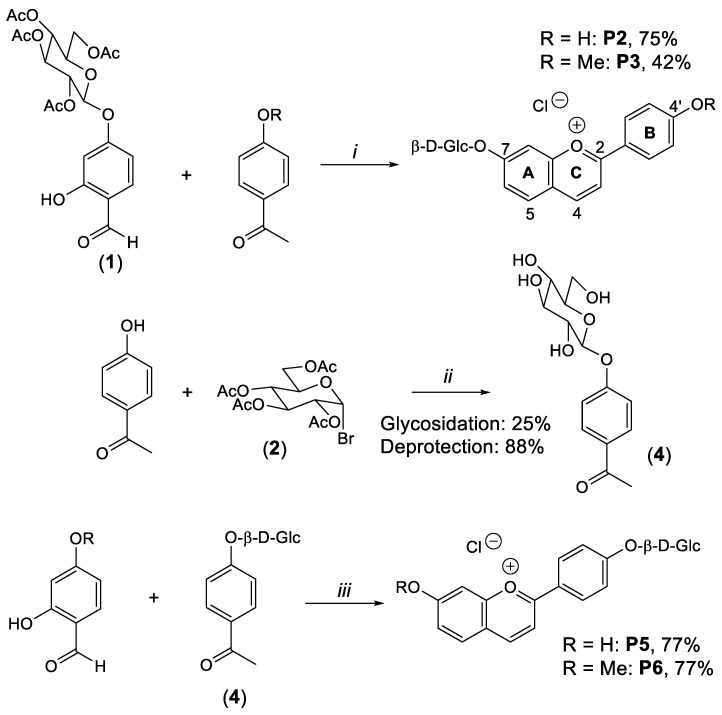
Chemical synthesis of pigments [13].

A summary of the chemical synthesis of pigments is presented in Scheme 2. 

### 4.2. Reagents

All starting materials were obtained from commercial suppliers. 4′-hydroxyacetophenone, 4′-methoxyacetophenone, 2, 4-dihydroxybenzaldehyde, 2-hydroxy-4-methoxybenzaldehyde, and chlorotrimethylsilane were purchased from Aldrich-Sigma (Saint-Quentin-Fallavier, France). 2, 4, 6-Trihydroxybenzaldehyde was purchased from Extrasynthese (Rhône, France). 3′,4′-Dihydroxyacetophenone and 4-(2′,3′,4′,6′-tetra-O-acetyl-b-D-glucopyranosyloxy)-2-hydroxybenzaldehyde (1) were prepared as reported previously [14].

TLC analyses were performed as described previously [13]. Purifications of intermediates were performed using column chromatography with silica gel 60 (40–63 mm, from Merck (Rahway, NJ, USA)). Pigments were purified from elution on C18 silica gel cartridges (bond elut from Varian (Palo Alto, CA, USA)).

^1^H and ^13^C NMR spectra were recorded on an Ascend^TM^ 400 Bruker apparatus at 400.18 MHz (^1^H) or 100.62 MHz (^13^C). Chemical shifts (d) are in ppm relative to tetramethylsilane using the deuterium signal of the solvent (CDCl_3_, CD_3_OD) for calibration. ^1^H-^1^H coupling constants (J) are in Hz. The ^1^H and ^13^C NMR spectra were published previously [13].

UPLC-MS analyses were completed using the parameters established previously [13]. H2O/HCO2H (99:1, *v*/*v*) (eluent A) and MeCN/H_2_O (60:40) + 1%HCO_2_H (eluent B) at a flow rate of 0.5 mL min^−1^ were used as the mobile phase. The elution program was as follows: 5–20% B (0–5 min), 20–100% B (5–10 min), 5–100% B (10–11 min), and 5% B (11–14 min). UV-vis absorption spectra were recorded on an Agilent 8453 diode array spectrometer equipped with a magnetically stirred quartz cell (optical path length—1 cm). The temperature in the cell was controlled by means of a water-thermostated bath at 25 °C.

HRMS analysis was carried out on a Qstar Elite mass spectrometer (Applied Biosystems SCIEX, Foster City, CA, USA). Mass detection was performed in the positive ESI mode.

UV-vis absorption spectra were recorded on an Agilent 8453 diode array spectrometer equipped with a magnetically stirred quartz cell (optical path length—1 cm). The temperature in the cell was controlled by means of a water-thermostated bath at 25 °C.

### 4.3. Cell Culture

The PC-3 (CRL-1435^TM^) prostate cancer cell line was obtained from the American Type Culture Collection (Manassas, VA, USA) and cultured in F12K cell culture medium supplemented with 10% fetal bovine serum (Wisent Inc., St-Bruno, QC, Canada) and penicillin/streptomycin sulfate (50 mg/L). Cells were incubated at 37 °C and 5% CO_2_.

### 4.4. Cell Viability Assay

Following treatments of PC-3 cells with increasing concentrations of the indicated molecules for 24 h, the cells were incubated with 0.2 mg/mL resazurin for 4 h at 37 °C. Then, cell viability was determined by measuring fluorescence (Ex = 550 nm, Em = 605, bandwidth = 10 nm).

### 4.5. Plasmids and Transfection

The pNFkB-luc (LR-2001), pCREB-luc (LR-2008), and pSOX5-luc (LR-2090) reporter plasmid constructs harboring six consensus regulatory elements for NFkB (5′-GGGAATTTCC-3′), three consensus regulatory elements for CREB (5′-TGACGTCA-3′), or four consensus regulatory elements for SOX (5′-TCAACAATCC-3′), respectively, were purchased from Signosis Inc. (Santa Clara, CA, USA). The SOX5 DNA regulatory elements of the pSOX5-luc plasmid can also be recognized by other members of the SOX family. Hence, this plasmid is referred to as pSOX-luc in the remainder of this manuscript. For the pNFkB-luc plasmid, the inserted sequence can be recognized by RELA, NFkB1, or NFkB2 according to the JASPAR database [41]. The human −670 to +92 bp *CDH1* promoter/luciferase reporter construct (#42083) was purchased from Addgene (Watertown, MA, USA). For transfections, PC-3 cells were plated in 96-well plates, incubated for 24 h, and transfected using polyethylenimine according to the method described previously [42]. After an incubation of 48 h, cells were treated with increasing concentrations of the indicated molecules for 6 h, followed by cell lysis and measurement of luciferase activity using a Varioskan luminometer (Thermo Scientific, Waltham, MA, USA).

### 4.6. In Silico Physicochemical Properties and Drug-likeness Evaluation

The SwissADME web tool [28] was used to assess the physicochemical properties of the synthesized compounds 16a-c and 17a, along with CDC (4) and CAPE (2) as references. Subsequently, the results were filtered based on the Lipinski rule of five, to estimate the potential bioavailability and drug-likeness [29].

### 4.7. Statistics

Experiments were repeated three or four times, and the data were presented as means ± S.E.M. Statistical analysis of the data was performed using one-way ANOVA followed by a Dunnett or Holm–Sidak multiple comparisons test using GraphPad Prism version 10.1.0 (GraphPad Software Inc., San Diego, CA, USA). *p* < 0.05 was considered significant.

## 5. Conclusions

Overall, among the molecules investigated in this study, the synthesized pigment P7 and its glycosylated analog P3 increase the activities of NFkB, CREB, and SOX transcription factors, leading to the upregulation of *CDH1* promoter activity in PC-3 prostate cancer cells. These changes in gene regulation may contribute to the inhibition of the epithelial-to-mesenchymal transition in cancer cells. Also, the natural pigment APN may reduce the viability of PC-3 prostate cancer cells by decreasing the activities of NFkB and SOX transcription factors.

## Figures and Tables

**Table 1:** Absorption, distribution, metabolism, and excretion (ADME) profile of the molecules of interest.

	Physicochemical Properties				Lipophilicity		Pharmacokinetics	
	MW (g/mol)	ROTB (n)	HBA (n)	HBD (n)	TPSA (Å)	CLogP_o/w_	GIA	BBBP
Rule	<500	≤10	<10	<5	≤140	<5	-	-
P1	290.70	1	4	3	73.83	0.26	High	No
P2	452.84	4	9	6	152.98	−1.23	Low	No
P3	436.84	4	8	5	132.75	−1.16	Low	No
P5	436.84	4	8	5	132.75	−0.84	Low	No
P6	450.87	5	8	4	121.75	−0.42	High	No
P7	274.70	1	3	2	53.60	0.91	High	Yes
APN	290.70	1	4	3	73.83	0.23	High	No

Abbreviations: BBBP, blood–brain barrier permeation; CLog Po/w, logarithm of compound partition coefficient between n-octanol and water; GIA, gastrointestinal absorption; HBA, hydrogen bond acceptors; HBD, hydrogen bond donors; MW, molecular weight; n, number; ROTB, rotatable bonds; TPSA, topological polar surface area.

## Data Availability

The original contributions presented in this study are included in this article. Further inquiries can be directed to the corresponding author.
